# FADU: a Quantification Tool for Prokaryotic Transcriptomic Analyses

**DOI:** 10.1128/mSystems.00917-20

**Published:** 2021-01-12

**Authors:** Matthew Chung, Ricky S. Adkins, John S. A. Mattick, Katie R. Bradwell, Amol C. Shetty, Lisa Sadzewicz, Luke J. Tallon, Claire M. Fraser, David A. Rasko, Anup Mahurkar, Julie C. Dunning Hotopp

**Affiliations:** a Institute for Genome Sciences, University of Maryland School of Medicine, Baltimore, Maryland, USA; b Department of Microbiology and Immunology, University of Maryland School of Medicine, Baltimore, Maryland, USA; c Department of Medicine, University of Maryland School of Medicine, Baltimore, Maryland, USA; d Greenebaum Cancer Center, University of Maryland School of Medicine, Baltimore, Maryland, USA; University of Pennsylvania

**Keywords:** bacteria, differential expression, operon, polycistronic transcripts, read count, software, transcriptome, transcriptomics

## Abstract

Quantification tools for RNA sequencing (RNA-Seq) analyses are often designed and tested using human transcriptomics data sets, in which full-length transcript sequences are well annotated. For prokaryotic transcriptomics experiments, full-length transcript sequences are seldom known, and coding sequences must instead be used for quantification steps in RNA-Seq analyses. However, operons confound accurate quantification of coding sequences since a single transcript does not necessarily equate to a single gene. Here, we introduce FADU (Feature Aggregate Depth Utility), a quantification tool designed specifically for prokaryotic RNA-Seq analyses. FADU assigns partial count values proportional to the length of the fragment overlapping the target feature. To assess the ability of FADU to quantify genes in prokaryotic transcriptomics analyses, we compared its performance to those of eXpress, featureCounts, HTSeq, kallisto, and Salmon across three paired-end read data sets of (i) Ehrlichia chaffeensis, (ii) Escherichia coli, and (iii) the *Wolbachia* endosymbiont *w*Bm. Across each of the three data sets, we find that FADU can more accurately quantify operonic genes by deriving proportional counts for multigene fragments within operons. FADU is available at https://github.com/IGS/FADU.

**IMPORTANCE** Most currently available quantification tools for transcriptomics analyses have been designed for human data sets, in which full-length transcript sequences, including the untranslated regions, are well annotated. In most prokaryotic systems, full-length transcript sequences have yet to be characterized, leading to prokaryotic transcriptomics analyses being performed based on only the coding sequences. In contrast to eukaryotes, prokaryotes contain polycistronic transcripts, and when genes are quantified based on coding sequences instead of transcript sequences, this leads to an increased abundance of improperly assigned ambiguous multigene fragments, specifically those mapping to multiple genes in operons. Here, we describe FADU, a quantification tool for prokaryotic RNA-Seq analyses designed to assign proportional counts with the purpose of better quantifying operonic genes while minimizing the pitfalls associated with improperly assigning fragment counts from ambiguous transcripts.

## Abstract (precis)

Most currently available quantification tools for transcriptomics analyses have been designed for human data sets, in which full-length transcript sequences, including the untranslated regions, are well annotated. In most prokaryotic systems, full-length transcript sequences have yet to be characterized, leading to prokaryotic transcriptomics analyses being performed based on only the coding sequences.

## INTRODUCTION

Differential expression transcriptomics analyses frequently involve the quantification of the number of paired-end reads, or fragments, that are overlapping each gene. Traditional quantification tools, such as featureCounts (1) or HTSeq (2), first require an alignment step, in which paired-end reads are aligned to a reference genome using tools such as Bowtie2 (3, 4), BWA (5), or HISAT2 (6). The subsequent quantification step uses the output alignment file in combination with a GFF/GTF annotation file to quantify the number of sequenced fragments that intersect the coordinates of each target gene. For transcriptomics experiments in which no whole-genome reference is available, a different subset of quantification tools was developed using *de novo* transcriptome assemblies, including the cufflinks suite of tools (7–10), RSEM (11), and eXpress (12). These tools bypass the need for a GFF/GTF annotation file and instead involve an alignment step in which fragments are mapped to reference transcript sequences instead of the whole genome. Most recently, alignment-free tools, such as kallisto (13) and Salmon (14), bypass the alignment step altogether and use raw sequencing fragments to directly quantify reference transcript sequences using pseudoalignments and k-mer-based counting approaches (13, 14).

All of these approaches work best with a well-annotated reference genome in which full-length transcript sequences have been identified. Each of the above-mentioned quantification tools was developed using human transcriptomics data sets as a template, and because of this, these tools are deficient when used to quantify genes for prokaryotic transcriptomics studies. Under ideal conditions, transcriptomic studies would quantify genes at the transcript level, but the lack of complete transcript annotations for most nonmodel eukaryotic and prokaryotic organisms forces transcriptomic analyses to be conducted at the coding sequence (CDS) level, failing to account for the untranslated regions (UTRs) of transcripts. This can be problematic in genomes with dense coding capacities and overlapping transcripts. It is especially problematic when analyzing prokaryotes due to the abundance of polycistronic transcripts from operons, with there being an estimated 630 to 700 operons in the >5,000 genes found in the Escherichia coli genome (15).

At the core of these problems is the method with which ambiguous fragments are quantified. Ambiguous fragments can be divided into two categories: (i) multimapping fragments, in which a fragment maps to multiple genomic regions equally, such as in the case of reads originating from paralogous genes, and (ii) multigene fragments, in which a fragment maps uniquely but overlaps multiple features. Each tool has a different method to quantify these types of ambiguous reads. By default, the genome alignment-based tools featureCounts and HTSeq fail to quantify multimapping fragments and mark them as ambiguous, while some transcript-alignment-based and alignment-free tools, such as cufflinks, eXpress, RSEM, kallisto, and Salmon, apply abundance estimation algorithms to assign partial counts. As an example, RSEM, eXpress, kallisto, and Salmon all apply an expectation maximization (EM) algorithm to ambiguous fragments, in which the number of fragments that unambiguously align to a target transcript is used to estimate the counts from ambiguous fragments originating from that target transcript (16).

In the case of quantifying operons in prokaryotic systems, difficulties often stem from the quantification of multigene fragments. By default, featureCounts will assign a multigene fragment to the feature that overlaps the majority of individual paired-end reads in a given fragment and, in the case of a perfect split, marks the read ambiguous (1). In comparison, HTSeq does not quantify any multigene fragments and instead marks them as ambiguous (2). Both of these approaches lead to an underestimation of the actual mapped reads for these genes and, by extension, an underestimation of gene expression. Tools such as cufflinks, eXpress, RSEM, kallisto, and Salmon again rely on abundance estimation algorithms to assign proportional counts for both multigene fragments.

**FIG 1:**
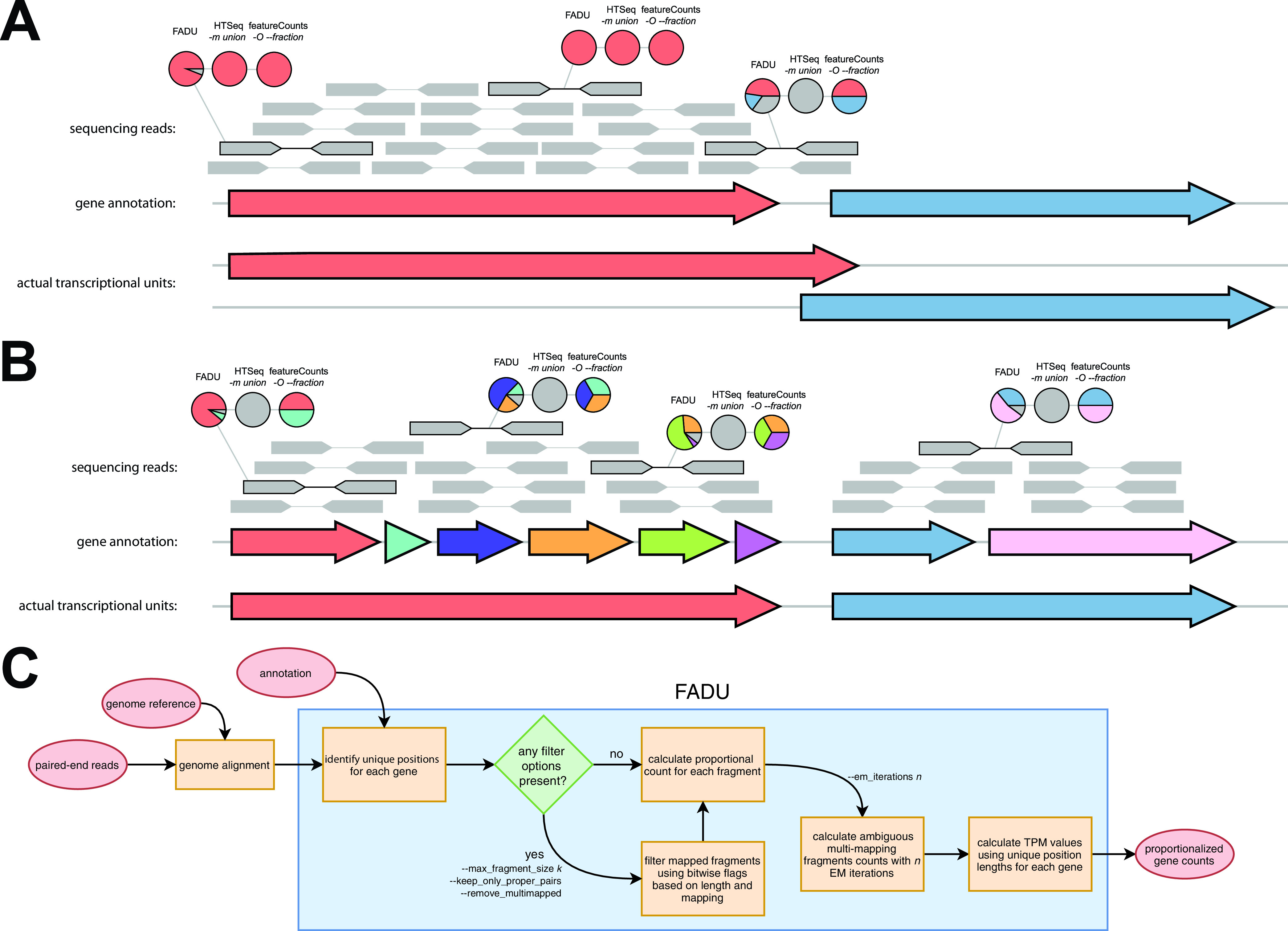
Implementation of FADU. (A) The workflow of FADU uses a BAM file and a GFF annotation file to identify proportional read counts for prokaryotic RNA-Seq analyses. (B) The implementation of FADU differs from those of other similar genome alignment-based quantification tools primarily in the quantification of ambiguous multigene fragments. The three sets of pie charts above a paired-end fragment display how the counts from the fragment are proportionally assigned to its overlapping genes. In the case of two overlapping genes, FADU accounts for only the unique portions of each gene and assigns a proportional count based on the length that the fragment overlaps the feature. (C) In the case of operons, FADU will assign proportional counts to the different genes based on the overlap between the mapping coordinates of the fragment and any overlapping genic features.

However, the strategies used by these tools are inherently flawed when quantifying prokaryotic genes. For featureCounts and HTSeq, multigene fragments occur frequently in operons. While both of these tools have alternative modes for quantification, in which counts are assigned to all overlapped features, this often leads to an overquantification of features in close proximity that are not transcribed together (Fig. 1A). When run with default settings, discarding these reads underestimates the abundance of operonic genes, especially smaller genes in the middle of operons (Fig. 1B). The tools cufflinks, eXpress, kallisto, RSEM, and Salmon all require a reference containing transcript sequences. Because of the absence of full-transcript prokaryotic annotations, CDSs must often be used instead, leading to the discarding of fragments that map primarily to the 5′ or 3′ regions of genes. Additionally, the absence of transcript annotations for operons further complicates the analysis. The use of CDSs as the units for quantification implies that each CDS is a unique observation, which is not the case for operons. For fragments that overlap multiple genes in an operon, this causes the fragment count to be improperly split into multiple genes when it should optimally be equally counted for all genes. Ideally, this issue would be solved by first identifying the full-length transcript sequences for prokaryotes using laboratory techniques such as 5′ and 3′ rapid amplification of cDNA ends (RACE) or direct RNA sequencing (RNA-Seq) preceding transcriptomics-based analyses, but this is currently not practical given the number of different prokaryotic systems being studied.

In this study, we developed FADU (Feature Aggregate Depth Utility), a quantification tool specifically designed for prokaryotic transcriptomics analyses, to address the shortcomings centered around the quantification step of prokaryotic transcriptomics. FADU uses an alignment file generated by aligning reads to a whole-genome assembly and handles ambiguous multigene fragments by proportionally assigning fragment counts. Given a multigene fragment, FADU assesses the proportion of the fragment that overlaps the nonunique positions of each of its overlapped features and assigns a proportional fragment count. By assigning proportional read counts, FADU avoids the pitfalls that other tools have in quantifying operonic genes while minimizing the errors derived from quantifying multigene fragments. Here, we describe the implementation of FADU and compare its performance and utility to those of the alignment-dependent quantification tools featureCounts, HTSeq, and eXpress and the alignment-free quantification tools kallisto and Salmon.

## RESULTS

### FADU implements a quantification method based on assigning proportional counts based on fragment overlap.

FADU was designed with the objective of addressing ambiguous multigene fragments. A multigene fragment is a fragment represented by a read pair whose mapping coordinates overlap multiple features, which occurs in dense coding regions and operons. To properly identify multigene fragments in the absence of robust and rigorous operon predictions, we designed FADU to function using a BAM file aligned to a reference genome.

For each paired-end fragment, FADU functions by assigning proportional counts based on the length of the different features that the fragment overlaps (Fig. 1C) as *F_c_* = *F_O_/F_L_*, where *F_c_* represents the proportional fragment count contribution for a given fragment or read for each genic feature, *F_O_* represents the number of bases that the fragment overlaps the unique positions of the genic feature, and *F_L_* represents the total length of the fragment. In the case of overlapping genes, no proportional count values will be derived from ambiguous positions. For reads that are unable to be processed as read pairs, *F_c_* is halved to prevent the contribution of discordant reads or singletons from overestimating the counts of a feature, as these discordant reads could be the result of potentially erroneous mappings. Following the assignment of uniquely mapping counts, using an expectation maximization (EM) approach (17) implemented similarly in other RNA-Seq quantification tools such as eXpress (12), kallisto (13), and Salmon (14), by default, FADU will derive counts from ambiguous multimapping fragments. After *n* EM iterations, transcripts per million (TPM) values for each genic feature are calculated using their unique positional lengths.

### Comparing the performance of FADU against other quantification tools using a simulated data set.

We compared the performance of FADU to those of the default modes of several different RNA-Seq quantification tools, including the genome-alignment-based quantification tools featureCounts (1) and HTSeq, the transcriptome-alignment-based quantification tool eXpress, and the alignment-free quantification tools kallisto and Salmon. In addition to the default modes for each of these tools, we also assessed the derived counts from some of these tools using options designed to optimize the quantification of prokaryotic RNA-Seq data sets. This includes the *-O* and *–fraction* options for featureCounts, which will quantify ambiguous multigene fragments by assigning either a full count value or a proportional count value derived from the number of features that a paired-end fragment overlaps, respectively. We also compared the performance of FADU to those of all three modes of HTSeq, *-m union*, *-m intersection-nonempty*, and *-m intersection strict*, along with *-m unique* with the option *–nonunique all* (2). Compared to HTSeq *-m union*, HTSeq *-m intersection-nonempty* is liberal in assigning multigene fragments. Given a multigene fragment, HTSeq *-m intersection-nonempty* takes the intersect of the genic features found at each nonempty position, and if only one genic feature is returned, a count is assigned to that genic feature. HTSeq *-m intersection-strict* is more conservative and takes the intersect of the genic features found at all positions rather than the nonempty positions, and again, if only one genic feature is returned, a count is assigned to that genic feature. Additionally, HTSeq *–nonunique all* functions similarly to featureCounts *-O* in that a full count value is assigned to all genes overlapped by a multigene fragment. For eXpress (12), we assessed the performances of the *-B 10* and *–no-bias-correct* options, which increases the number of EM iterations used in deriving counts from ambiguous fragments and ignores sequence-specific biases, respectively. Finally, for Salmon, we assessed the performances of the *–validateMapping* and *–allowDovetail* options to better quantify genic features with lengths shorter than the paired-end fragment length.

For these comparisons, we generated a simulated RNA-Seq data set for Escherichia coli K-12 substrain MG1655 using transcript annotations generated from operon predictions from OperonDB (18). Using each of the different quantification methods, we derived count values from the simulated RNA-Seq data set using CDS annotations, instead of the predicted operon annotations, to best assess the performance of each quantification method in the absence of operon predictions. Using Polyester (19), four samples were simulated, consisting of two conditions consisting of two replicates each, to simulate a basic minimum differential expression analysis. Totals of 256 and 300 transcripts were simulated to be significantly over and underexpressed, respectively, using the operon predictions from OperonDB. Of these transcripts, 184 and 116 are operonic transcripts, resulting in there being 595 and 678 significantly up- and downregulated CDSs, respectively.

**FIG 2:**
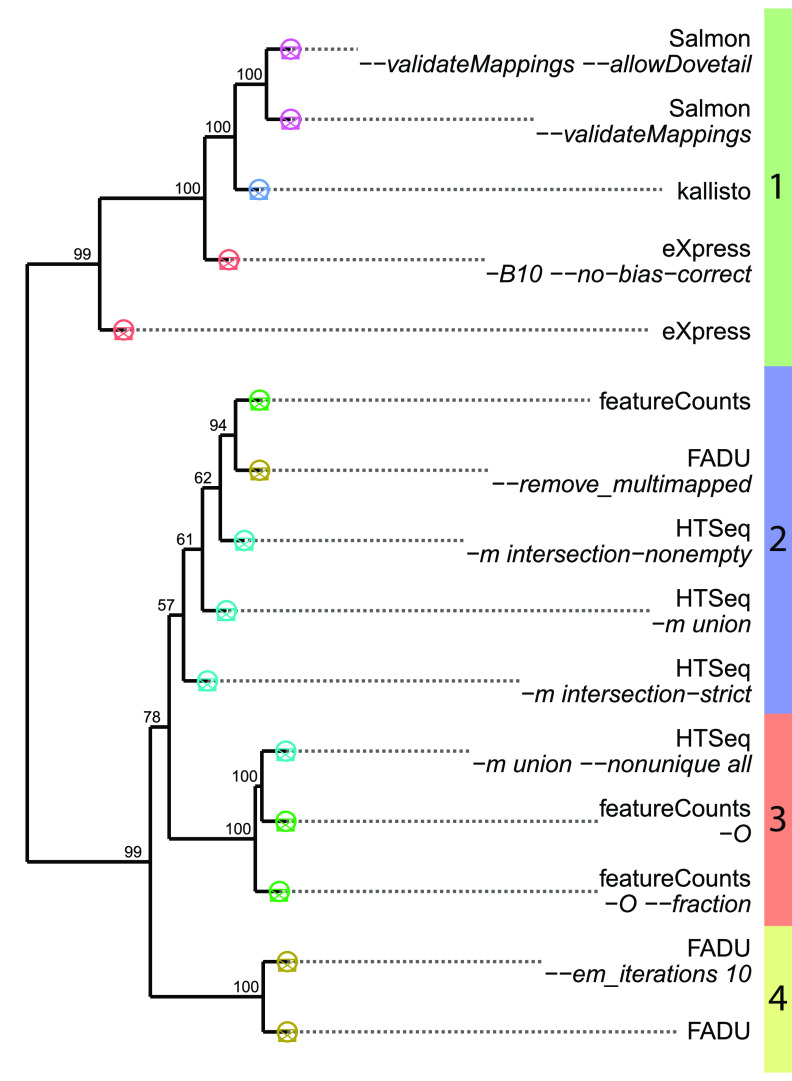
Performance of FADU in a simulated differential expression analysis. A 100-bootstrap dendrogram was generated using the counts obtained from 9 different RNA-Seq quantification methods on a simulated E. coli data set. The points at the edges of the dendrogram are colored to represent the different tools corresponding to each method. The colored bar at the edge of the dendrogram represents the different clusters of quantification tools.

Using TPM values derived from each quantification method, we conducted a hierarchical clustering analysis to divide the methods into four distinct clusters (Fig. 2). The first cluster consists of all methods in which a transcriptome-based reference is used, including all three alignment-free quantification methods (green bar on the right of the dendrogram). The second cluster (blue bar) consists of the more conservative genome-alignment-based quantification methods, in that counts are often not derived from multigene fragments as they are instead marked as ambiguous and ignored. In contrast, the third cluster (orange bar) consists of the genome-alignment-based quantification methods that are more liberal in assigning counts from multigene fragments, in which a count value is given to all features that a multigene fragment overlaps. The fourth cluster (yellow bar) contains both methods of FADU, which use an EM approach to quantify ambiguous multimapping fragments while assigning proportional counts for multigene fragments. With FADU, excluding the assignment of multimapping fragments with the *–remove_multimapping* option yields counts most similar to those of the conservative genome-based quantification methods (cluster 2), setting FADU apart as the only genome alignment quantification method that uses an EM-based method to assign multimapping fragments.

**FIG 3:**
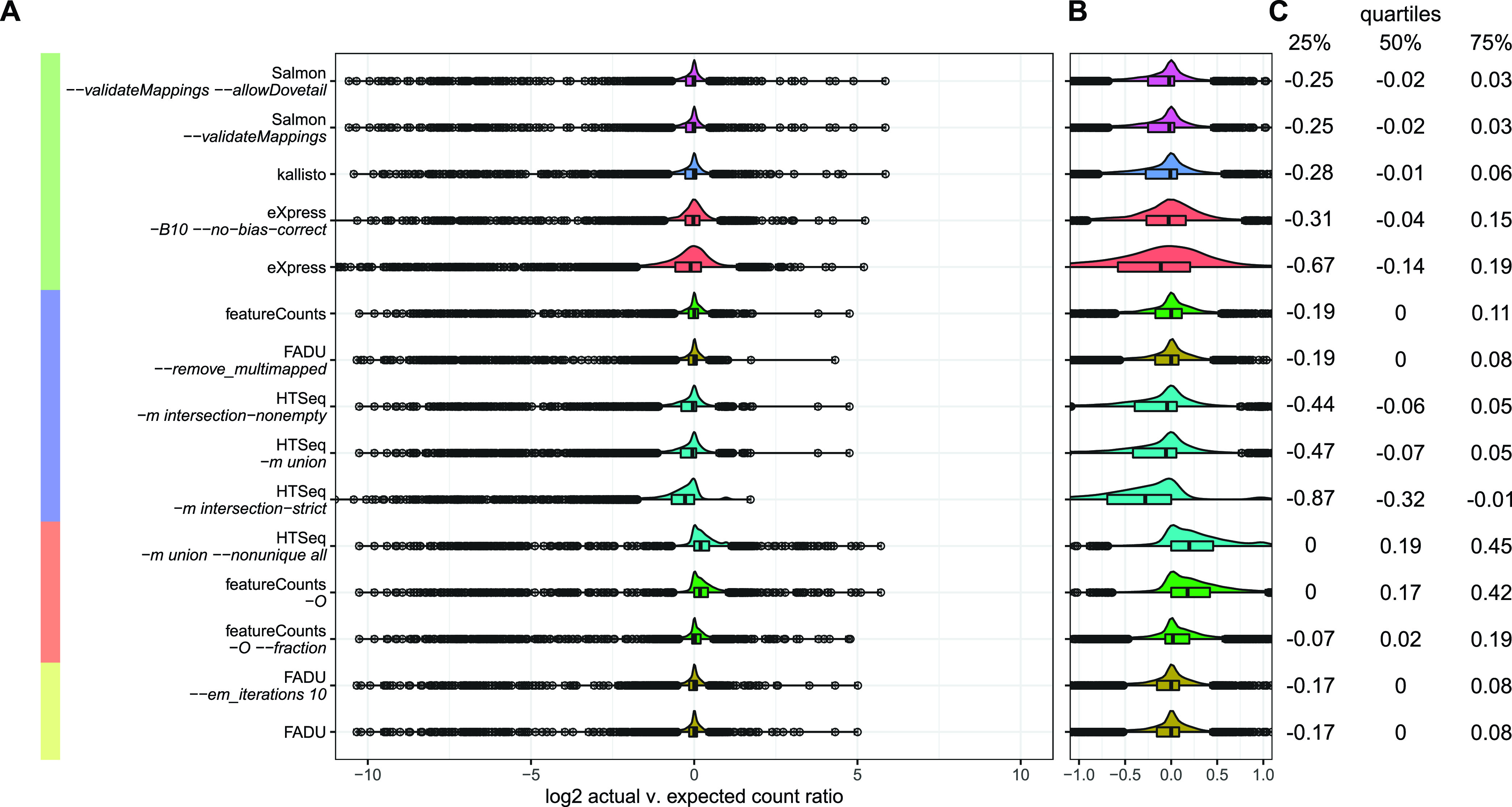
Accuracy of FADU in a simulated RNA-Seq data set. (A) For each quantification method, a log_2_ ratio was calculated for the counts obtained for each gene in the simulated data set versus the actual counts expected from the simulated data set. The distributions for each of these methods would ideally be normal and centered at zero. (B) Zoomed-in version of the distribution generated from the log_2_ ratios. (C) The interquartile ranges for each of the distributions show the precision of each method.

Differential expression analyses were conducted using two differential expression tools, DESeq2 (20) and edgeR (21), using the counts derived from each quantification method. Using DESeq2 and edgeR, all methods, with the exception of eXpress run with default settings, were at least able to correctly detect approximately half of the simulated differentially expressed CDSs (Fig. 3b and c). With edgeR, all methods except eXpress with default settings were able to correctly detect ∼200 more differentially expressed genes than with DESeq2, although the number of false-positive differentially expressed genes detected was also greater.

Of the different methods, the cluster consisting of the genome-alignment-based quantification methods that more liberally assign counts from multigene fragments (cluster 1) was able to consistently detect the most differentially expressed genes. However, these methods also consistently have the highest false-positive rates (DESeq2, 0.41 to 0.54%; edgeR, 1.36 to 1.49%) as they incorrectly identify more nondifferentially expressed genes as differentially expressed (Tables 1 and 2). Of the conservative genome-alignment-based methods (cluster 2), FADU *–remove_multimapping* detects the most differentially expressed genes while retaining a false-positive rate similar to those of the other tools. As the tools in this cluster become more conservative in assigning multigene fragments, fewer differentially expressed genes are able to be detected, partly because more are being excluded from the default DESeq2 and edgeR minimum-expression filters (Tables 1 and 2). As an example, HTSeq *-m intersection-strict* is the most conservative quantification tool in this study for deriving counts from multigene fragments. As such, it excludes the most genes from these analyses, which include 40 incorrectly assigned differentially expressed genes. The alignment-free quantification methods perform similarly to the FADU methods that implement an EM algorithm for assigning counts from multimapping fragments. However, using DESeq2, FADU is able to detect ∼10 genes more as differentially expressed while having a slightly higher false-positive rate, while with edgeR, only FADU *–em_iterations 10* outperforms the alignment-free methods in detecting differentially expressed genes while keeping a similar false-positive rate (Tables 1 and 2). Ten EM iterations were chosen as convergence typically occurs within 10 iterations for these prokaryotic genomes and annotations (see Fig. S1 in the supplemental material).

**TABLE 1:** DESeq2 differential expression statistics^*a*^

Quantification method	Cluster	No. of correct DE genes	No. of correct NDE genes	No. of missed DE genes	No. of false-positive DE genes	Detection rate (%)	False-positive rate (%)	No. of DE genes excluded from expression threshold	No. of NDE genes excluded from expression threshold
Salmon *–validateMappings* *–allowDovetail*	1	673	3,156	587	3	53.41	0.09	22	5
Salmon *–validateMappings*	1	674	3,156	586	3	53.49	0.09	22	5
Kallisto	1	673	3,150	587	9	53.41	0.28	58	4
eXpress *-B10 –no-bias-correct*	1	599	3,152	661	7	47.54	0.22	158	14
eXpress	1	290	3,119	970	40	23.02	1.27	163	17
featureCounts	2	694	3,154	566	5	55.08	0.16	97	15
FADU *–remove-multimapped*	2	703	3,156	557	3	55.79	0.09	92	15
HTSeq *-m intersection-nonempty*	2	656	3,156	604	3	52.06	0.09	156	17
HTSeq *-m union*	2	645	3,158	615	3	51.19	0.09	178	20
HTSeq *-m intersection-strict*	2	607	3,142	653	1	48.17	0.03	357	42
HTSeq *-m union –nonunique all*	3	770	3,142	490	17	61.11	0.54	51	6
featureCounts *-O*	3	776	3,142	484	17	61.59	0.54	55	6
featureCounts *-O –fraction*	3	735	3,146	525	13	58.33	0.41	52	6
FADU *–em_iterations 10*	4	686	3,149	574	10	54.44	0.32	52	10
FADU	4	684	3,152	576	7	54.29	0.22	53	9

a DE, differentially expressed; NDE, nondifferentially expressed.

**TABLE 2:** edgeR differential expression statistics

Quantification method	Cluster	No. of correct DE genes	No. of correct NDE genes	No. of missed DE genes	No. of false-positive DE genes	Detection rate (%)	False-positive rate (%)	No. of DE genes excluded from expression threshold	No. of NDE genes excluded from expression threshold
Salmon *–validateMappings* *–allowDovetail*	1	946	3,115	314	44	75.08	1.39	98	5
Salmon *–validateMappings*	1	946	3,115	314	44	75.08	1.39	98	5
Kallisto	1	947	3,116	313	43	75.16	1.36	141	4
eXpress *-B10 –no-bias-correct*	1	831	3,137	429	22	65.95	0.70	237	14
eXpress	1	53	3,143	1,207	16	4.21	0.51	292	17
featureCounts	2	936	3,135	324	24	74.29	0.76	169	15
FADU *–remove-multimapped*	2	974	3,127	286	32	77.30	1.01	153	15
HTSeq *-m intersection-nonempty*	2	903	3,128	357	31	71.67	0.98	267	17
HTSeq *-m union*	2	909	3,119	351	40	72.14	1.27	295	20
HTSeq *-m intersection-strict*	2	802	3,134	458	25	63.65	0.79	499	42
HTSeq *-m union –nonunique all*	3	1,006	3,113	254	46	79.84	1.46	76	6
featureCounts *-O*	3	1,008	3,116	252	43	80.00	1.36	80	6
featureCounts *-O –fraction*	3	1,000	3,112	260	47	79.37	1.49	109	6
FADU *–em_iterations 10*	4	955	3,122	305	37	75.79	1.17	112	10
FADU	4	947	3,125	313	34	75.16	1.08	111	9

10.1128/mSystems.00917-20.1 FIG S1 EM convergence on iteration. The convergence of the EM algorithm was measured by taking the absolute value of the difference between the current iteration and the previous iteration for each gene, where the index iteration was 0 and the final iteration was 100. The sum across all genes was plotted for the current iteration for sample 1 of the simulated data (A), *E. chaffeensis* (SRA accession number SRR1188323) (B), E. coli (SRA accession number SRR2601722) (C), and *Wolbachia* endosymbiont *w*Bm (SRA accession number SRR5192555) (D). Download FIG S1, PDF file, 0.08 MB. Copyright © 2021 Chung et al. 2021 Chung et al. This content is distributed under the terms of the Creative Commons Attribution 4.0 International license.

For each quantification method, we calculated a log_2_ ratio of the quantification-method-derived counts to the simulated counts to assess the accuracy and precision of the counts derived from each of the quantification methods. The ideal quantification method would have a distribution with a log_2_ ratio value centered at around zero, indicating that most counts derived for each genic feature are similar to the simulated values, and a low interquartile value, indicating high precision in the quantification-method-derived counts (Fig. 3A to C). The transcriptome-based quantification tools of the first cluster all have distributions that are slightly left-skewed, indicating that most features are being undercounted. While this difference is very slight for the kallisto- and Salmon-based methods, the high interquartile range for both eXpress methods indicates low precision in quantifying features in this simulated prokaryotic RNA-Seq data set. In the second cluster, all three HTSeq methods run with the three different modes are similarly left-skewed, again indicative of many features being undercounted, due to fewer count values being derived from multigene fragments. The default featureCounts method and FADU *–remove_multimapped* are the most accurate and precise of the conservative genome-based quantification methods in that they both have low interquartile ranges and their ratio distributions are centered around zero. The increased precision of featureCounts relative to HTSeq lies in how each method quantifies multigene fragments. By default, featureCounts will assign a multigene fragment to the feature that maps to the feature overlapping the majority of the individual reads in a paired-end fragment (1), while HTSeq derives no counts from all multigene fragments and instead marks them all as ambiguous (2).

The methods in the third cluster, consisting of the genome-alignment-based quantification methods that liberally derive counts from multigene fragments, all have right-skewed distributions, indicating that most features are being overcounted (Fig. 3A to C). For a multigene read mapping in an operonic structure, the ideal quantification method should assign an equal count value to all overlapped genic features. While this may result in an inflation of read counts relative to the number of reads mapped, the expression values would be more accurately quantified. However, because of the dense nature of prokaryotic genomes, it becomes difficult to determine whether a multigene fragment is representative of an operonic transcript or overlaps multiple genes due to the high coding density of prokaryotic genomes. The right-skewed nature of all three quantification methods in this cluster is a consequence of overinflating counts due to an overabundance of overlapping or close-proximity nonoperonic genes in the E. coli genome. The fourth cluster consists of both FADU methods that assign counts from multimapping fragments using an EM algorithm. Of all the analyzed methods, both FADU methods run with EM iterations have the lowest interquartile range and a distribution centered at zero, indicating that they most accurately quantify coding sequences in the absence of operon annotations for this simulated data set.

### Comparing the performance of FADU against those of other quantification tools.

To assess the performance of each of the quantification methods when faced with variation arising from real data, we compared the performances of the same quantification methods on three different transcriptomics data sets consisting of (i) paired-end reads from a standard (i.e., not-strand-specific) library constructed from Escherichia coli RNA, (ii) paired-end reads from a standard library constructed from Ehrlichia chaffeensis RNA, and (iii) paired-end reads from a strand-specific library constructed from RNA isolated from the *Wolbachia* endosymbiont strain *w*Bm from Brugia malayi. For the alignment-based quantification tools, reads were mapped to a genomic reference for FADU, featureCounts, and HTSeq or a coding sequence reference for eXpress. For the alignment-free quantification tools kallisto and Salmon, reads were directly quantified using a coding sequence reference. Across all three data sets, the counts obtained using FADU are correlated with those obtained with the five other quantification tools (Fig. S2).

10.1128/mSystems.00917-20.2 FIG S2 Comparing fragment counts from FADU versus other quantification tools. Across the three prokaryotic transcriptomic data sets of unstranded E. coli (a) and *E. chaffeensis* (b) and stranded *w*Bm (c), a density plot was generated by plotting the fragment counts obtained using the default settings of FADU against the counts obtained using 12 other quantification methods. Download FIG S2, PDF file, 0.8 MB. Copyright © 2021 Chung et al. 2021 Chung et al. This content is distributed under the terms of the Creative Commons Attribution 4.0 International license.

**FIG 4:**
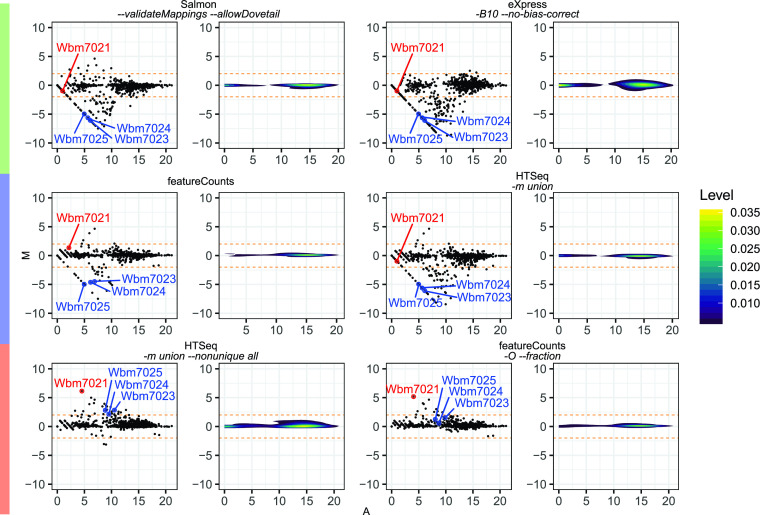
MA plots comparing *w*Bm fragment counts obtained using FADU against those obtained using other quantification tools. The fragment counts for the stranded *w*Bm data set obtained using FADU were compared to the counts obtained using methods representative of the different clusters of quantification methods. The *x* axis denotes the mean average from the two compared counts (A), while the *y* axis denotes the log_2_ ratio of the two compared counts (M) as a scatterplot (left) and a density plot (right). The horizontal orange dotted lines on each plot are drawn at log_2_ ratio values of 2 and −2. Points with a log_2_ ratio greater than 2 and less than −2 were defined as genes counted differently by FADU relative to its counterpart tool.

For pairwise comparisons between FADU and two representative quantification methods from each of the hierarchical clusters in Fig. 2, MA plots were constructed (Fig. 4) by calculating the mean average of the log_2_ counts (A) and the log_2_ ratio counts (M) for each gene. Genes with a log_2_ count ratio of ≥2 or ≤2 were defined as being significantly differentially counted between FADU and the compared quantification method. There are 135 unique CDSs in the *w*Bm data set across the five pairwise comparisons that had higher counts by FADU (Fig. 4). Of the analyzed methods, eXpress -*B10 –no-bias-correct* contains most of these genes, with 122 CDSs (90.3%) having higher counts with FADU. Additionally, Salmon *–validateMappings –allowDovetail*, featureCounts, and HTSeq *-m union* have 68, 67, and 29 CDSs that have higher counts with FADU. In comparison, HTSeq *-m union –nonunique all* and featureCounts *-O –fraction* have at most 2 genes with higher counts using FADU.

A total of 36 unique CDSs were identified as having lower counts using FADU than using one of the six analyzed quantification methods. (Fig. 4). Of the analyzed quantification methods, FADU has the fewest counted genes compared to HTSeq *-m union –nonunique all* and featureCounts *–O –fraction*. As both of these tools derive counts from multigene fragments, FADU produces fewer counts for 28 (77.8%) and 13 (36.1%) CDSs than HTSeq *-m union –nonunique all* and featureCounts *–O –fraction*, respectively. FADU compared to all other methods has fewer counts for <10 CDSs.

**FIG 5:**
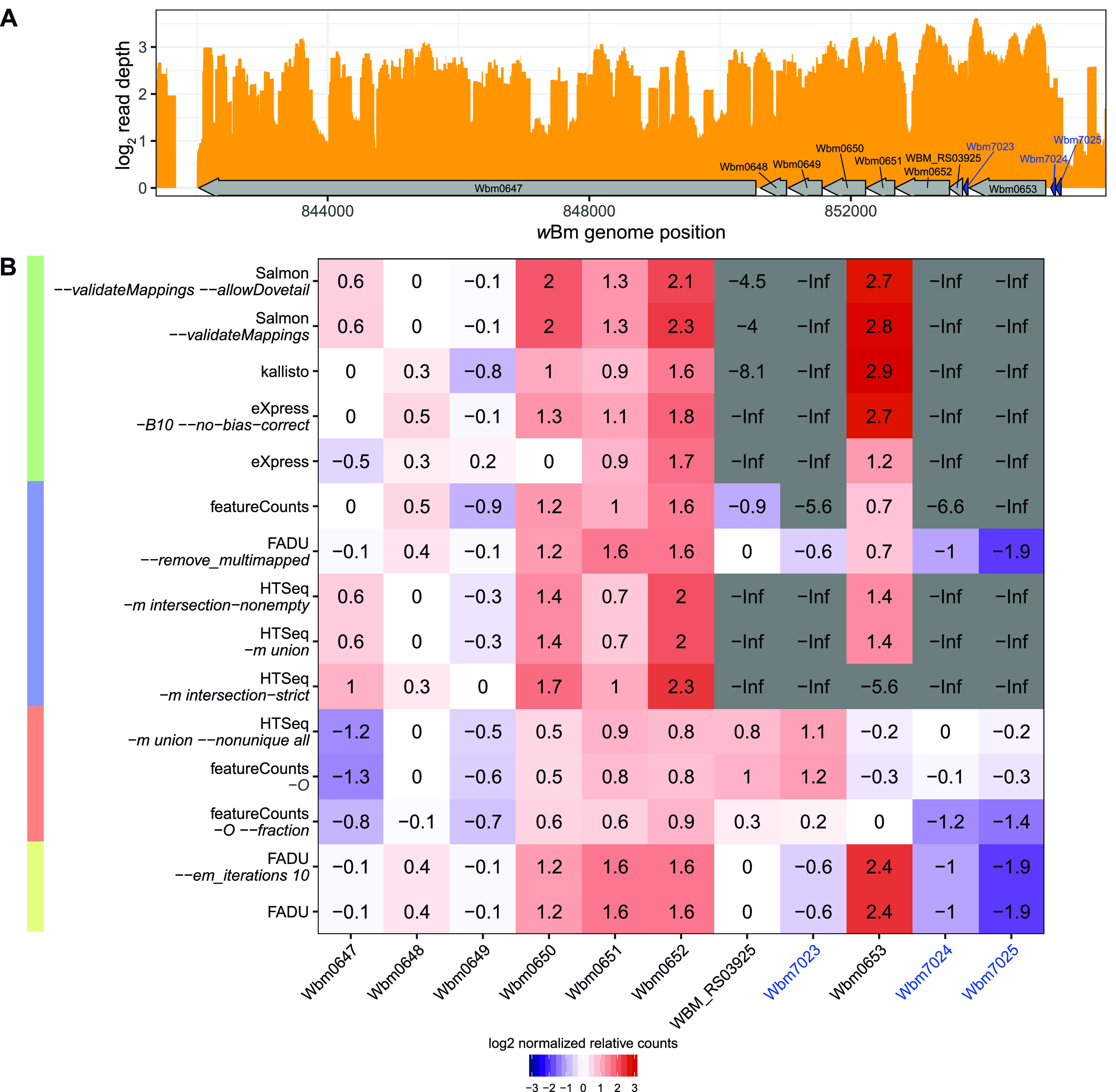
Performance of quantification tools for deriving counts for operons. (A) Using the *w*Bm stranded RNA-Seq data set, the log_2_ read depth (orange) was plotted for part of an operon containing 11 genes. Genes labeled and marked in blue are all significantly undercounted (log_2_ count ratio of less than −1), as assessed in Fig. 4. (B) For each quantification tool, the read depth per base pair was calculated for all 11 genes displayed and divided by the median read depth per base pair across the operon for each quantification mode to obtain a normalized relative count value. The log_2_-transformed normalized relative count values are displayed in the individual cells of the table. -Inf is used to denote when the quantification tool returned “0” for the gene such that the ratio cannot be log transformed. Because the 11 genes are transcribed together, we would expect the normalized values obtained for each of the 11 genes to be ∼0. Normalized values in red cells indicate that the gene has a higher count value than the other operonic genes, while blue cells indicate that the gene has a lower count value than the other operonic genes. Tools that discard ambiguous reads spanning two features in close proximity have a tendency to undercount the smaller genes in operons, such as Wbm7023, Wbm7024, and Wbm7025.

The MA analysis highlights differences in how ambiguous multigene fragments are quantified. High counts with FADU are often found relative to quantification methods that conservatively quantify genes in multigene fragments, while low counts by FADU are often relative to the genome-based quantification methods that derive counts from genes with ambiguous multigene fragments. In total, we observed 24 CDSs with higher counts by FADU than by eXpress *–B10 –no-bias-correct*, Salmon *–validateMappings –allowDovetail*, featureCounts, and HTSeq *-m union*. Of the 24 genes, 3 are within a putative 11-gene operon: Wbm7023, Wbm7024, and Wbm7025 (Fig. 5A). Within this 11-gene region, based on the depth track, empirically, all genes should have roughly the same count values. Within this operon, we divided the fragment counts for each gene by their respective gene lengths to obtain a fragment count per base pair value for each gene. For each quantification method individually, these fragment count per base pair values were normalized by dividing by the median fragment counts per base pair for all genes in this 11-gene operonic region (Fig. 5B). Normalized values that are higher than 3 or lower than −3 indicate that the gene is significantly over- or undercounted relative to the rest of the operon.

For Wbm7023, Wbm7024, and Wbm7025, despite all three genes having a fragment depth that appears to be similar to that of the rest of the putative operon (Fig. 5A), only the FADU-based methods and HTSeq *-m union –nonunique all*, featureCounts *-O*, and featureCounts *-O –fraction* properly assign counts to all three. The difficulty in assigning counts to the three genes stems from their close proximity to adjacent genes combined with their individual small sizes. Similarly, the smaller size of WBM_RS03925 leads to undercounting with eXpress, kallisto, and Salmon. In the case of transcriptome-based aligners, the small sizes of the three genes make it difficult for eXpress to identify reads that mostly map to each of the genes. Similarly, the smaller size makes it difficult for kallisto and Salmon to identify unique k-mers for these genes, leading to both genes being undercounted by the k-mer-based tools (Fig. 5B).

**FIG 6:**
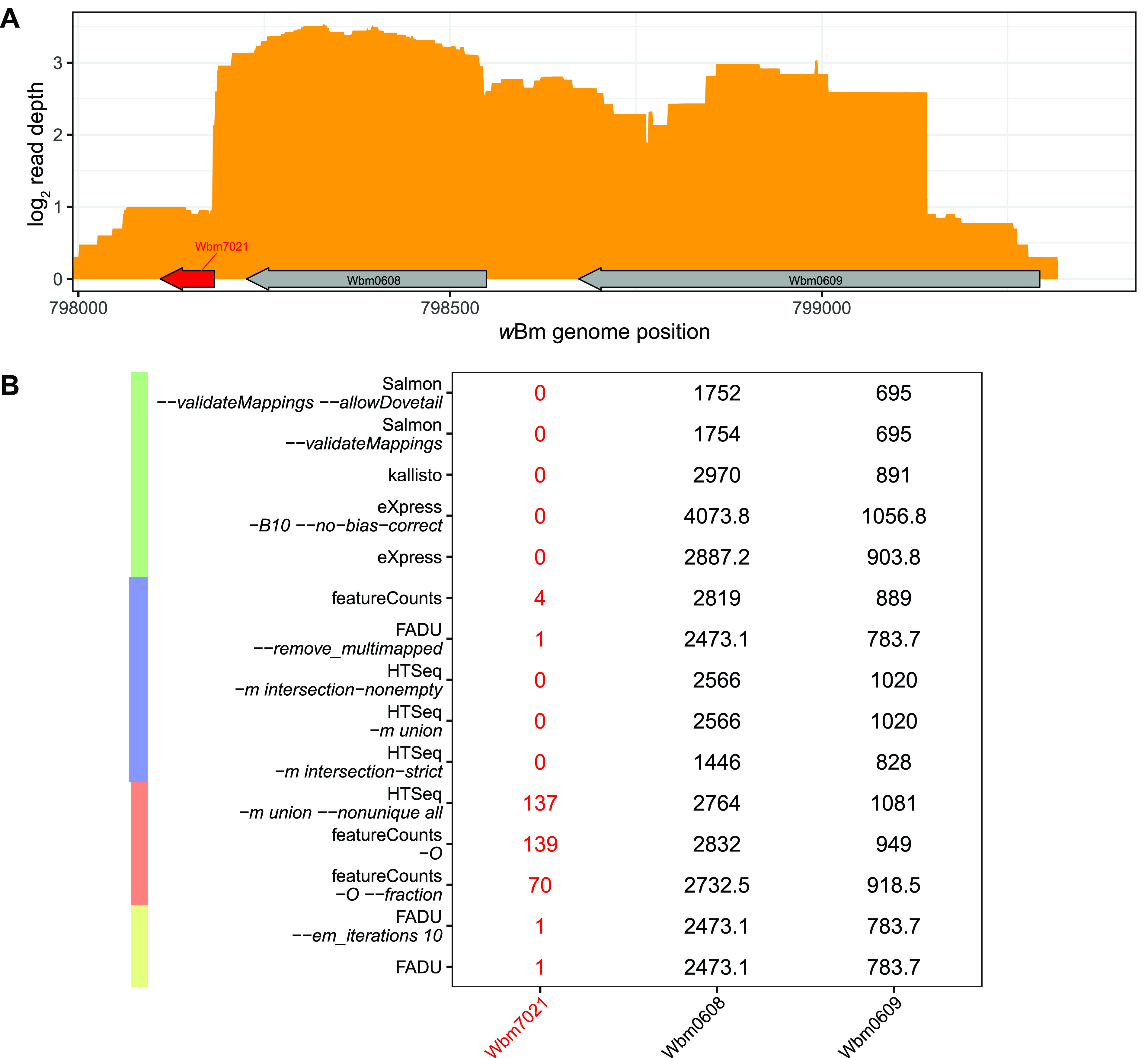
Improper quantification of fragments that span multiple features. (A) Using the *w*Bm stranded RNA-Seq data set, the log_2_ read depth (orange) was plotted across the *w*Bm gene Wbm7023 and its adjacent genes Wbm0608 and Wbm0609. (B) The counts for the three genes as determined by each of the quantification methods were calculated. The counts assigned to Wbm7023 are all derived from fragments that also map to the 3′ end of Wbm0608. Despite the *w*Bm annotation lacking UTRs, these reads likely originate from the 3′ UTR of Wbm0608, indicating that most if not all the reads assigned to Wbm7023 are erroneous.

By deriving counts from ambiguous multigene fragments, FADU, HTSeq *-m union –nonunique all*, featureCounts *-O*, and featureCounts *-O –fraction* all run the risk of incorrectly assigning counts in instances where fragments originate from monocistronic transcripts that are in close proximity, particularly in the case of gene-dense genomes rather than operons. Because each of these tools assigns fragment counts to all overlapped features, the counts from a fragment that originates from one gene can be mistakenly assigned to another gene overlapped by the fragment. While this is erroneous in the case of monocistronic transcripts, scenarios such as this are difficult to avoid without well-annotated transcripts and without undercounting smaller operonic genes. However, the proportional fragment counts assigned by FADU minimize the errors from such instances. As an example, the *w*Bm gene Wbm7021 is in close proximity to Wbm0608 (Fig. 6A), separated by 44 bp, such that reads from the unannotated 3′ UTR of Wbm0608 are erroneously being counted for Wbm7021. FADU mitigates this issue by only assigning proportional fragment counts based on the percentage of the fragment’s length that overlaps a feature. While most quantification methods obtain almost no reads for Wbm7021, HTSeq *-m union –nonunique all*, featureCounts *-O*, and featureCounts *-O –fraction* obtain 137, 139, and 70 read counts for Wbm7021, respectively (Fig. 6B). In comparison, all methods of FADU limit this error and obtain only 1 read count for Wbm7021. Collectively, by assigning proportional counts, FADU is better able to quantify operonic genes while mitigating the issues stemming from quantifying multigene fragments.

### Timing and memory benchmarks.

**FIG 7:**
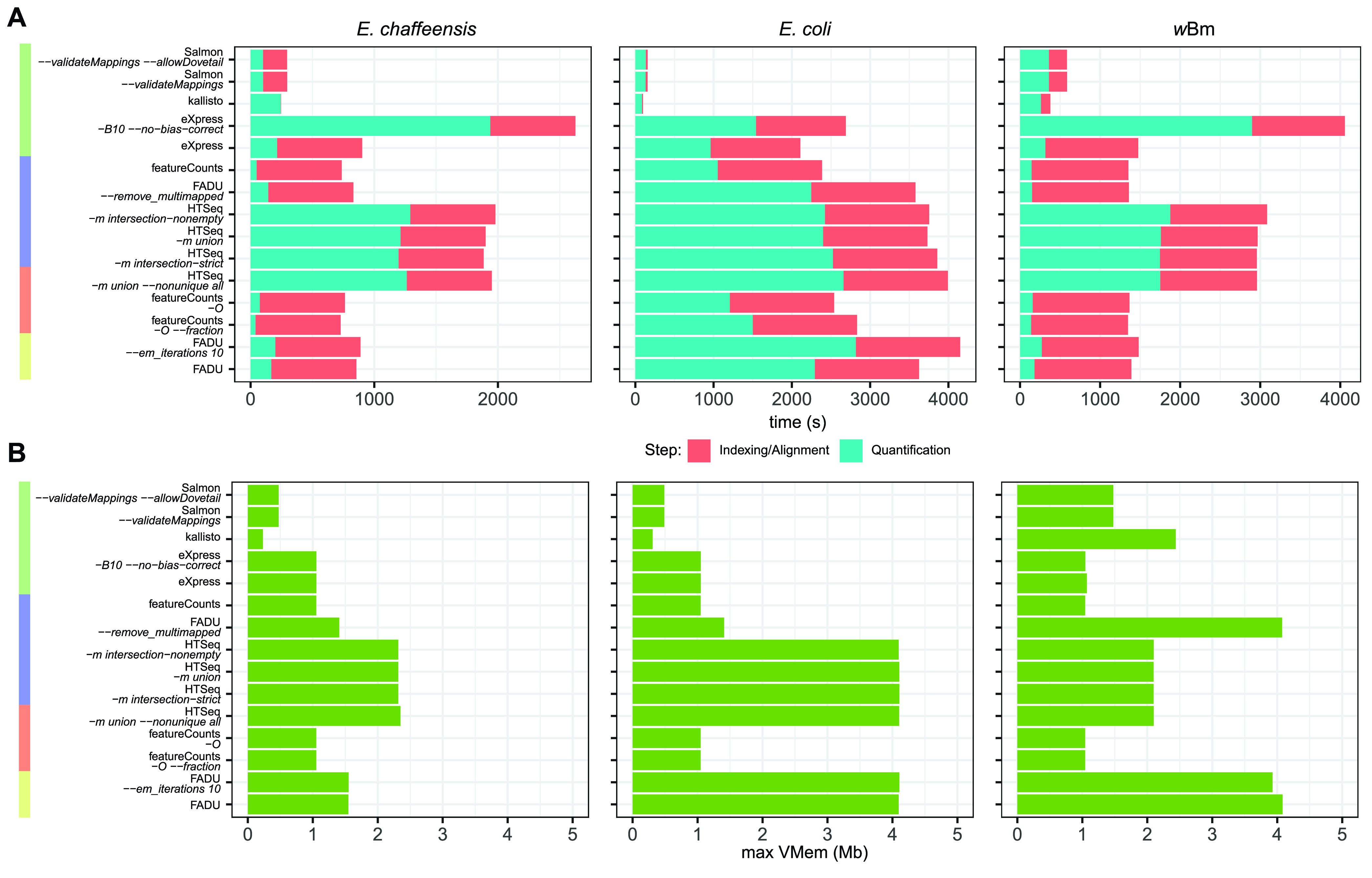
FADU timing and memory benchmarking. For the unstranded E. coli and *E. chaffeensis* and the stranded *w*Bm data sets, the wall clock speed for the indexing and/or alignment steps and quantification steps using 4 threads (A) along with the maximum memory (max Vmem) used (green) (B) were recorded for all quantification methods analyzed.

We compared the speed (Fig. 7A; Fig. S3) and memory usage (Fig. 7C) of FADU to those of the 10 other quantification modes for *E. chaffeensis*, E. coli, and *w*Bm data sets run with 1 and 4 threads. For the alignment-based quantification tools eXpress, FADU, featureCounts, and HTSeq, the times for alignment and quantification were individually recorded, while for the alignment-free quantification tools kallisto and Salmon, the times for indexing and quantification were individually recorded. Across all three data sets analyzed, the alignment-free quantification tools kallisto and Salmon perform the fastest and use the least memory, as would have been expected from the methodology. The speed of FADU is either higher than or comparable to those of the other alignment-based quantification tools for the *E. chaffeensis* and *w*Bm data sets (Fig. 7A). However, when used to analyze the E. coli data set, FADU and HTSeq have longer run times than eXpress and featureCounts. When comparing the maximum memory usage between the different tools, FADU has memory requirements comparable to those of the other alignment-based quantification tools (Fig. 7B).

10.1128/mSystems.00917-20.3 FIG S3 FADU timing and memory benchmarking. For the unstranded E. coli and *E. chaffeensis* and the stranded *w*Bm data sets, the wall clock speed for the indexing and/or alignment steps (red) and quantification steps (blue) using 1 thread was recorded for all quantification methods analyzed. Download FIG S3, PDF file, 0.8 MB. Copyright © 2021 Chung et al. 2021 Chung et al. This content is distributed under the terms of the Creative Commons Attribution 4.0 International license.

## DISCUSSION

In an ideal transcriptomics differential expression analysis, full-length transcript annotation of the reference organism should be used for the alignment and/or quantification steps. In cases where full-length transcripts are available, we believe that ambiguous multigene fragments should be able to be assigned with confidence using abundance estimation strategies such as the EM algorithm (17). However, in cases where full-length transcripts are not available, CDSs are typically used instead. When CDSs are used, considerations need to be made regarding the shortcomings of quantifying ambiguous multigene fragments, especially in the context of prokaryotic systems, in which operonic structures and dense coding regions could be present.

Without knowledge of complete transcript sequences, it becomes impossible to determine whether an ambiguous multigene fragment stems from an operonic transcript or overlaps two genes in close proximity that are independent transcriptional units. While operon databases and prediction software are available, they are not comprehensive, and the gold standard for determining the sequences of full-length transcripts is still through laboratory-based studies such as 5′ and 3′ RACE. While advances in long-read sequencing will eventually lead to native-strand RNA sequencing (22), in which the sequences of full-length transcripts can be obtained easily, current technologies are lacking a robust method to easily annotate full-length nonpolyadenylated transcripts and, as a result, operons.

In situations where full-length transcript sequences are unavailable, mapping directly to transcript references will fail to quantify a subset of reads that originate primarily from untranslated regions instead of the coding sequence. For cases such as this, genome references perform better for deriving counts from noncoding sequence reads. By using the precise mapping information provided by a genome-based alignment, quantification tools can better infer whether a fragment overlaps a target gene. However, as we have shown, ambiguous reads are often either too conservatively or too liberally assigned, resulting in an under- or overestimation of transcript expression, respectively. FADU was designed for the purpose of optimizing the available alignment information for systems in which full-length transcript annotations are unavailable and CDS-based counting is confounded by operons and/or a high coding density, both of which are significant issues in prokaryote genomes. However, FADU is not splice aware and is thus not suitable for eukaryotic genomes with spliced genes. But by assigning proportional read counts, FADU maximizes the quantification of operonic genes while minimizing the false overquantification of nonoperonic genes in close proximity to one another.

## MATERIALS AND METHODS

### Implementation.

FADU was written entirely using the Julia programming language v1.0 (23) and uses the BioAlignments.jl and GenomicFeatures.jl packages. GenomicFeatures.jl is used to parse out record information from GFF files and determine overlaps between alignments and features. BioAlignments.jl is used to quickly parse record information from the BAM file. FADU was tested and benchmarked in the UNIX environment.

FADU first creates an interval tree-based data structure of the feature annotation (GFF3) input file consisting of the sequence identification (ID), the leftmost coordinate, the rightmost coordinate, the strand, and the feature metadata. This data structure is used to construct a set of all nonoverlapping coordinates per strand, defined as a genomic position that is not overlapped by two or more recorded features. If the BAM alignment input file is unstranded, the set of overlapping coordinates per attribute ID will be strand agnostic.

The BAM file is read in one alignment record at a time. For each record, validation steps are performed to ensure that the record is mapped, is a primary record, and exceeds the specified minimum mapping quality (default score of 10). If the option *–remove_multimapping* alignments is enabled, records whose “NH” attribute exceeds a value of 1 are also removed. Next, the record is assessed as to whether or not it is part of a fragment by taking into account read pair information. In order to classify a read as part of a fragment, both the template length of the read pair (default, <1,000 bp) and the 0-by-2 bitflag of each record are assessed. Records that do not meet the qualifications to be processed as “fragments” are instead processed as “reads.” For records classified as fragments, only one of the reads of the pair is kept because the coordinate information of the mate pair can be inferred. If the option to keep only properly paired reads is enabled, only records that can be classified as fragments will be kept.

After validation, each record is classified as either a fragment or a read for downstream processing. Each record is used to create an interval tree-based data structure consisting of the record reference sequence name, the leftmost and rightmost coordinates of the fragment or read, the strand of the fragment or read, and a designation of fragment or read. Once a sufficient number of these data structures is read into memory (the default chunk size is 10,000,000), the overlaps for each alignment record to the specified annotation feature type are processed. If the option *–remove_multimapping* alignments is disabled, alignment records that are multimapped are saved to be processed after all the uniquely mapped records are processed. The overlaps for the multimapped reads are then processed, but the counts overlapping each feature are adjusted via the EM algorithm (17). For each iteration of the EM algorithm, the contribution of a multimapped record’s overlap to a given annotation feature’s count total is adjusted by the relative abundance of each overlapping feature’s total counts for uniquely mapped records only.

Once all records have been processed, total counts for every feature ID for the specified attribute type are calculated and used to calculate normalized TPM values for each feature ID. For each feature ID, five tab-delimited fields are written to file: (i) feature ID, (ii) length of nonoverlapping coordinates, (iii) number of alignments to overlap the feature, (iv) total fractionalized alignment counts for the feature, and (v) TPM count for the feature.

### Update equations.

For each paired-end fragment, FADU functions by assigning proportional counts based on the length of the different features that the fragment overlaps (Fig. 1C), where *F_c_* represents the proportional fragment count contribution for a given fragment or read for each genic feature, *F_O_* represents the number of bases that the fragment overlaps the unique positions of the genic feature, *F_L_* represents the total length of the fragment, and

$$F_{c}=\frac{F_{O}}{F_{L}}$$

For *f* (feature [e.g., a gene]), *F* (all features), *N_f_* (total counts for feature *f*), *N_F_* (total counts for all features present), and *a_f_* (estimated relative abundance for feature *f* among all features *F*), then

$$N_{f}=\sum F_{c}$$

$$a_{f}=\frac{N_{f}}{N_{F}}$$

For *r* (each record for a fragment [e.g., a pair of reads]), *R* (all alignment records), *r*,*f* (a single feature mapping to record *r*), *r*,*F* (all features mapping to record *r*), *N_r_*_,_*_f_* (count for record *r* aligning to feature *f*), *N_r_*_,_*_F_* (total counts for all features that align to record *r* [including counts where the record aligns to other features]), and *a_r_*_,_*_f_* (relative abundance for feature *f* that mapped to record *r*), then

$$a_{r,f}=\frac{N_{f}}{N_{r,F}}$$

$$\overset{\;}{\underset{f\in F}{\sum }}a_{r,f}^{\;}=1$$

For the EM algorithm, only multimapping records are considered. For *Rmm* (all multimapping alignment records) and ${\bar{N}}_{\mathrm{Rmm},f}$ (total counts for feature *f* from all multimapping records in *Rmm*), then

$${\bar{N}}_{\mathrm{Rmm},f}=\underset{r\in \mathrm{Rmm}}{\sum }a_{r,f}N_{r,f}$$

For *k* (EM iteration cycle number), *K* (maximum number of cycles), *N_f_*_,0_ (counts for a given feature not including multimapped records), ${\bar{N}}_{R,f,k}^{\;}$ (adjustment counts for feature *f* based on all multimapped records *R* in EM iteration cycle *k*), and *N_f_*_,_*_k_* (updated counts for a feature for EM iteration cycle number *k*), then

$$N_{f,k}=N_{f,0}+{\bar{N}}_{\mathrm{Rmm},f,k}\text{ for }k=\text{1, 2, }\ldots \;K$$

All values are recalculated using *N_f_*_,_*_k_* to update abundance values for each cycle.

### Quantification method analyses of the simulated data set.

The strand-specific simulated RNA-Seq data set for Escherichia coli K-12 substrain MG1655 (GenBank accession number U00096.3) was generated using Polyester v1.9.7 (19), using an annotation generated from operon predictions from OperonDB (18). Four samples were simulated, consisting of two conditions consisting of two replicates each, for conducting differential expression analysis. Totals of 256 and 300 transcripts were simulated to be significantly over- and underexpressed using the operon predictions from OperonDB. For the subsequent quantification steps, genes from the simulated data set were quantified using the original CDS annotations. Simulated reads were aligned using HISAT2 v2.1.0 with the options *–X 1000 –no-spliced alignment -k 200* to either the target genome or transcriptome. Genes were quantified using FADU v1.7 run with default settings and the *–remove_mulitmapping* and *–em_iterations 10* options; eXpress v1.5.1 (12) run with default settings and the *-B10* and *–no-bias-correct* options; featureCounts (1) v1.6.4 run with default settings and the *–fraction* and *-O* options; HTSeq v0.11.0 (2) run with each of the *-m union*, *-m intersection-nonempty*, and -m *intersection-strict* options and *–nonunique all* options; kallisto v0.46.1 (13) run with default settings; and Salmon v1.1.0 (14) run with default settings and the *–allowDovetail* and *–validateMappings* options. Hierarchical clustering analyses were conducted using pvclust v2.0-0 using a correlation distance parameter and average cluster method. Differential expression analyses were conducted using DESeq2 (20) and edgeR (21), using their respective recommended minimum expression filters of a total of 10 read counts across all samples and a cutoff of 5 counts per million (CPM) in the sample in the data set with the fewest reads sequenced. For DESeq2, differentially expressed genes were identified with *cooksCutoff=T* and a false discovery rate (FDR) of <0.05, while for edgeR, differentially expressed genes were identified using glmQLFit and an FDR of <0.05.

### Quantification tool comparisons on actual data sets.

*E. chaffeensis* Arkansas (GenBank accession number NC_007799.1), E. coli O127:H6 strain E2348/69 (GenBank accession number NC_011601.1), and *w*Bm (GenBank accession number NC_006833.1) data sets were downloaded from the SRA, using SRAtoolkit v2.9.0, under SRA accession numbers SRX485438, SRX1322474, and SRX2508248, respectively. The paired-end data sets were each aligned to the genomes or transcriptomes of their respective organisms using HISAT2 v2.1.0 with the options *–X 1000 –no-spliced alignment -k 200*. In the case of *w*Bm, reads were aligned to a combined reference consisting of the *w*Bm genome/transcriptome along with the Brugia malayi (WormBase version WBPS9) genome/transcriptome to minimize erroneous mappings from *Wolbachia-Brugia* lateral gene transfer reads (24, 25). Genes were quantified for each data set using FADU v1.7 run with default settings and the *–remove_mulitmapping* and *–em_iterations 10* options; eXpress v1.5.1 (12) run with default settings and the *-B10* and *–no-bias-correct* options; featureCounts (1) v1.6.4 run with default settings and the *–fraction* and *-O* options; HTSeq v0.11.0 (2) run with each of the *-m union*, *-m intersection-nonempty*, and -*m intersection-strict* options and *–nonunique all* options; kallisto v0.46.1 (13) run with default settings; and Salmon v1.1.0 (14) run with default settings and the *–allowDovetail* and *–validateMappings* options.

MA plots were generated between FADU and the default modes of eXpress, featureCounts, HTSeq, kallisto, and Salmon. For each plot, the mean average of the log_2_ counts (A) was calculated and plotted against the log_2_ ratio of counts (M).

### Data availability.

Three data sets were used in all analyses consisting of RNA-Seq paired-end data from standard, nonstranded libraries originating from *E. chaffeensis* and E. coli and stranded libraries from *w*Bm. The sequencing reads for the three data sets can be found in the NCBI Sequence Read Archive under the following accession numbers: SRX485438, SRX1322474, and SRX2508248, respectively. Additional scripts and commands used for analyses and benchmarking are available at https://github.com/IGS/FADU.
