# Supplementary figures and images for: FADU: a Quantification Tool for Prokaryotic Transcriptomic Analyses
Source: mSystems. 2021 Jan 12;6(1):e00917-20. doi: 10.1128/mSystems.00917-20 (PMC7901478; doi:10.1128/mSystems.00917-20)

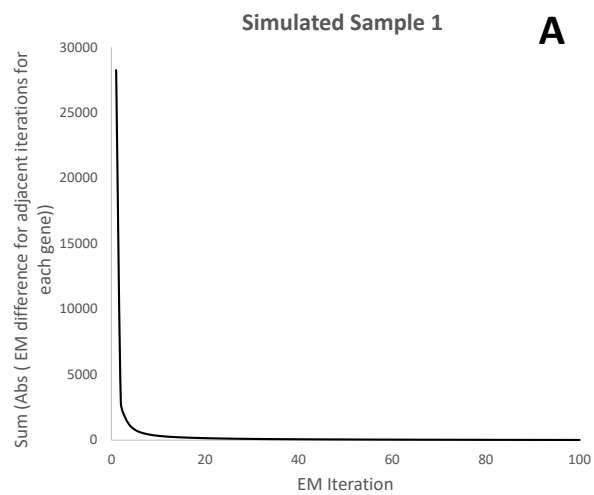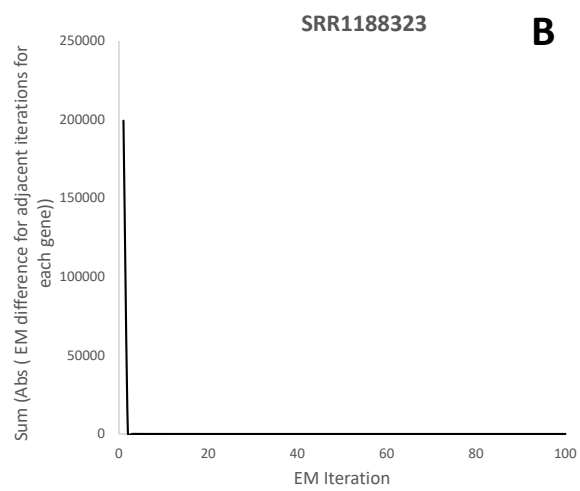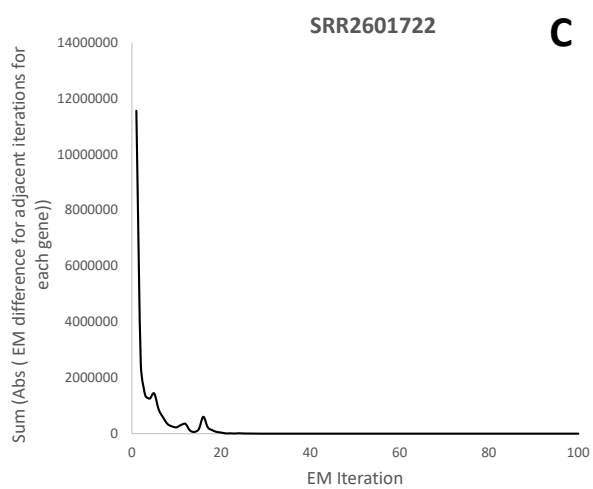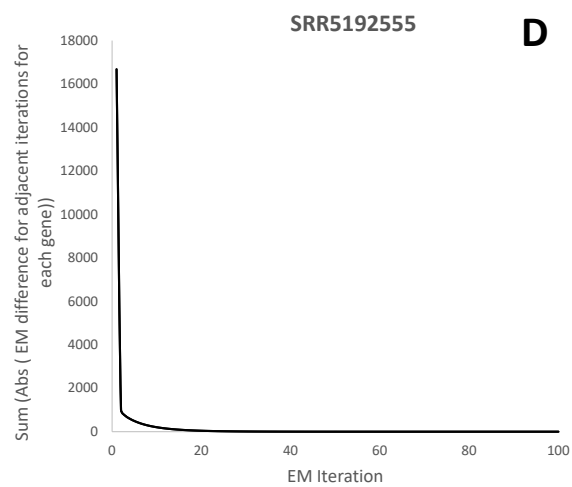

Supplement: FIG S1 [file mSystems.00917-20_sf001.pdf]

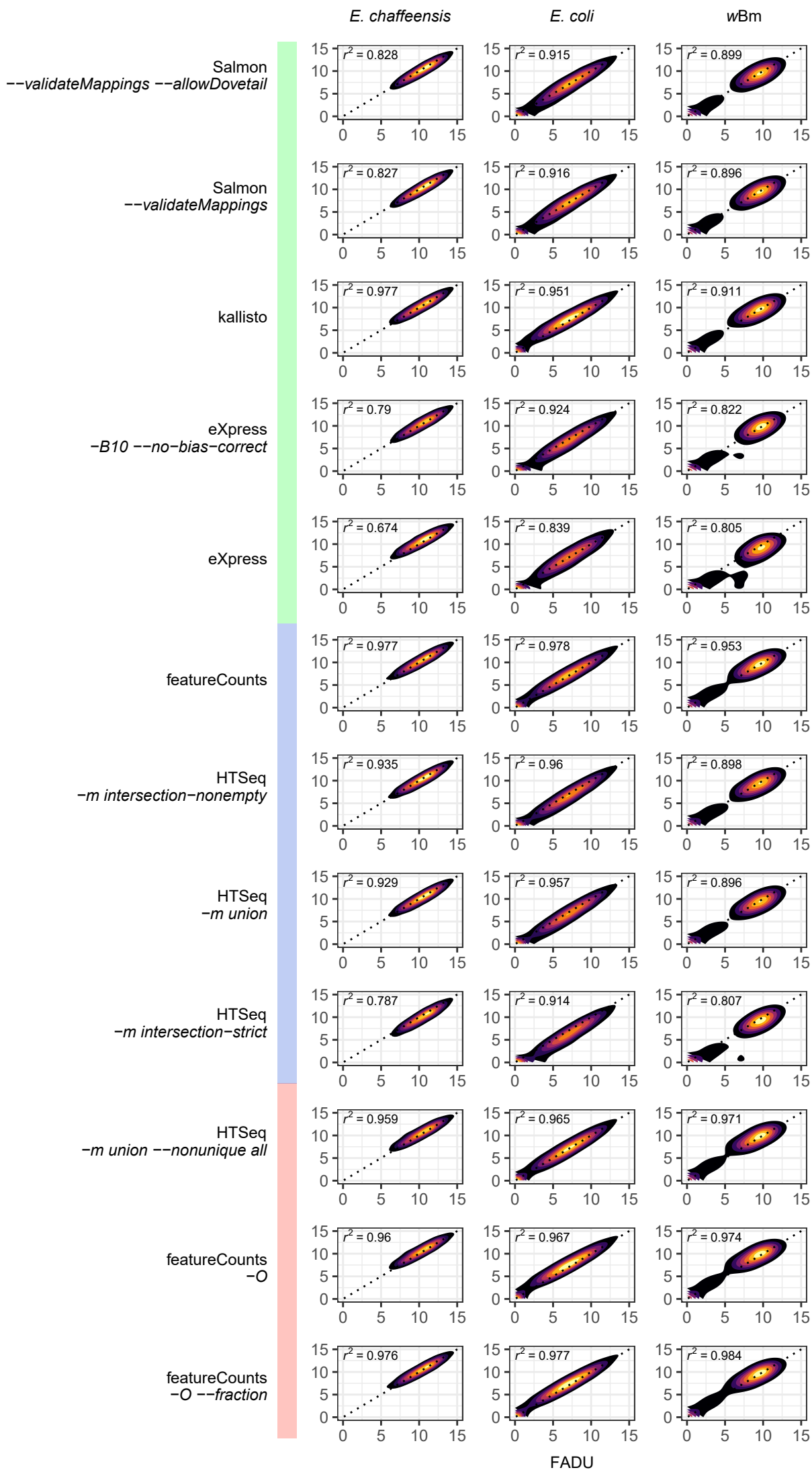

Supplement: FIG S2 [file mSystems.00917-20_sf002.pdf]

A

*E. chaffeensis**E. coli*

wBm

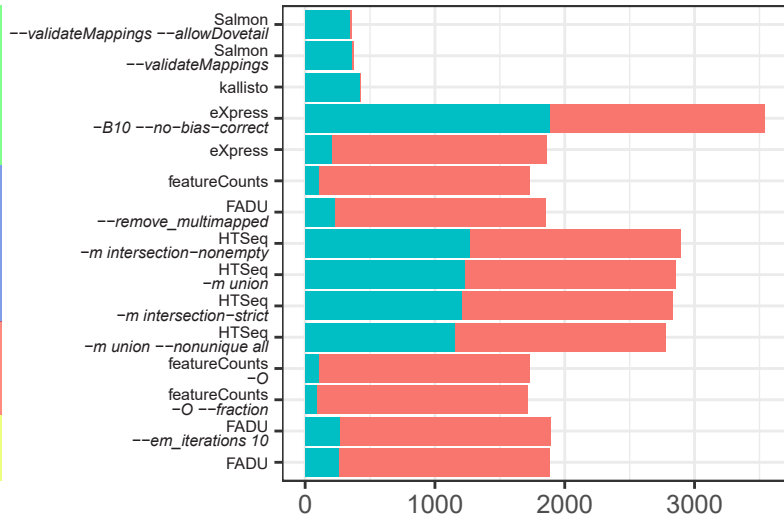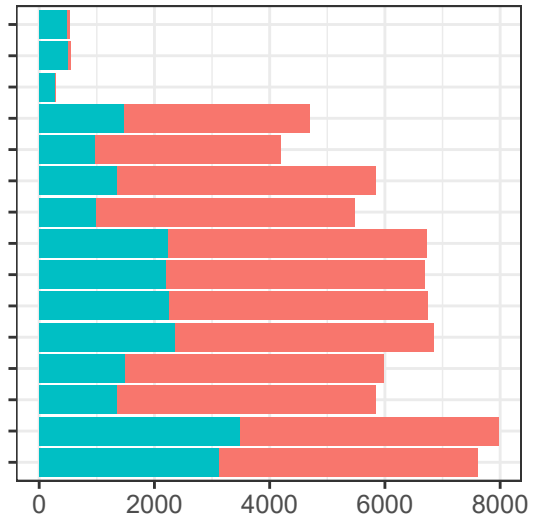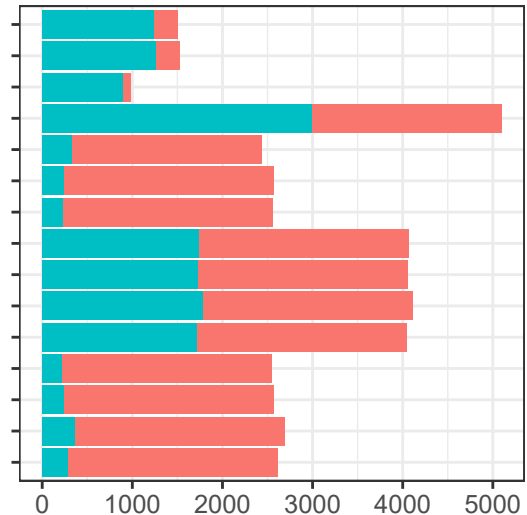

Step: Indexing/Alignment Quantification

Supplement: FIG S3 [file mSystems.00917-20_sf003.pdf]
